# Volatile Organic Compounds Emissions from *Luculia pinceana* Flower and Its Changes at Different Stages of Flower Development

**DOI:** 10.3390/molecules21040531

**Published:** 2016-04-22

**Authors:** Yuying Li, Hong Ma, Youming Wan, Taiqiang Li, Xiuxian Liu, Zhenghai Sun, Zhenghong Li

**Affiliations:** 1 Research Institute of Resource Insects, Chinese Academy of Forestry, Kunming 650224, China; liyuy_ing@163.com (Y.L.); hortscience@163.com (H.M.); wanyouming@126.com (Y.W.); li_taiqiang@163.com (T.L.); lxx205@126.com (X.L.); 2 School of Gardening, Southwest Forestry University, Kunming 650224, China; sunzhenghai1978@163.com

**Keywords:** *Luculia*, volatile organic compound, flower development, SPME-GC-MS, houseplant, floral scent

## Abstract

*Luculia* plants are famed ornamental plants with sweetly fragrant flowers, of which *L. pinceana* Hooker, found primarily in Yunnan Province, China, has the widest distribution. Solid phase microextraction-gas chromatography-mass spectrometry (SPME-GC-MS) was employed to identify the volatile organic compounds (VOCs) emitted from different flower development stages of *L. pinceana* for the evaluation of floral volatile polymorphism. Peak areas were normalized as percentages and used to determine the relative amounts of the volatiles. The results showed that a total of 39 compounds were identified at four different stages of *L. pinceana* flower development, including 26 at the bud stage, 26 at the initial-flowering stage, 32 at the full-flowering stage, and 32 at the end-flowering stage. The most abundant compound was paeonol (51%–83%) followed by (*E*,*E*)-α-farnesene, cyclosativene, and δ-cadinene. All these volatile compounds create the unique fragrance of *L. pinceana* flower. Floral scent emission offered tendency of ascending first and descending in succession, meeting its peak level at the initial-flowering stage. The richest diversity of floral volatile was detected at the third and later periods of flower development. Principal component analysis (PCA) indicated that the composition and its relative content of floral scent differed throughout the whole flower development. The result has important implications for future floral fragrance breeding of *Luculia*. *L. pinceana* would be adequate for a beneficial houseplant and has a promising prospect for development as essential oil besides for a fragrant ornamental owing to the main compounds of floral scent with many medicinal properties.

## 1. Introduction

*Luculia* Sweet is a small genus of the family Rubiaceae, consisting of about five types of small trees or shrubs. Three species occur in China, amongst which *Luculia pinceana* Hooker, found primarily in Yunnan Province at altitudes between 600 and 3000 m has the widest distribution in Nature [1,2,3]. *Luculia* can be easily recognized by its compact and long-term blooming (usually April to November) inflorescences with white to pink, sweetly fragrant flowers with extremely long corolla tubes. On account of the above-mentioned advantages, *Luculia* plants have been widely introduced around the world. Studies in the past are often concentrated on its reproductive system, distyly, molecular biology and gametophyte development [1,2,3,4,5,6,7]. In addition, as a traditional medicinal and ornamental plant, research work is underway concerning its cultivation and breeding.

Floral fragrance is one of the most important characteristics of ornamental plants or cut flowers, because it may affect people’s health and mood [8]. Thus, the study of the composition of floral scent and the breeding of new ornamental plant cultivars with sweet-smelling or pleasant fragrance have become new trends of the development of plant breeding nowadays [9]. Although *L. pinceana* possess the features of long-term blooming, sweet fragrance and has great potential in serving as an indoor plant, research on its floral scent has not been carried out. In this work, the volatile component emissions from different flower development stages of *L. pinceana* were analyzed by solid phase microextraction coupled with gas chromatography-mass spectrometry in order to build a foundation for future work on *Luculia* floral fragrance breeding programs. Furthermore, the results could provide a reference for determining whether *Luculia* species are suitable for indoor cultivation and extraction of essential oil.

## 2. Results and Discussion

### 2.1. Identification of Scent Components

Thirty-nine VOCs were identified, representing more than 99% of the total emission of the flowers. These volatiles grouped by their biochemical synthesis pathways [10] were described in Table 1. A total of 18 same volatile compounds were shared at four different stages of flower development of *L. pinceana*. Within these compounds, the main aroma-active one was paeonol followed by (*E,E*)-α-farnesene, cyclosativene, and δ-cadinene. These compounds might dominate the flavor for *L. pinceana*. For instance, paeonol has a specific odour; (*E*,*E*)-α-farnesene has a woody and sweet odour; δ-Cadinene gives thyme, medicine and wood odour [11]. As one of these components, γ-muurolene has a smell of herb, wood and spice; methyl salicylate has a flavor with peppermint aroma. The two compounds ranked second in relative content of VOCs at the full-flowering stage and the end-flowering stage separately, so they could also influence the floral aroma.

The idea that *L. pinceana* has medicinal properties goes back hundreds of years in China [12]. Some of volatile compounds from flowers are pharmacologically active compounds. For example, paeonol has several interesting biological activities, and it has been used as an anti-inflammatory, analgesic, antioxidant, antidiabetic, and acaricidal agent [13,14]; cyclosativene demonstrates strong anti-inflammatory, expectorant, antifungal effect [15]; γ-muurolene and δ-cadinene have antifungal properties. Despite the fact that essential oils are seldom encountered in the Rubiaceae [16], *L. pinceana* would have a lot of potential for essential oil extraction according to the *L. pinceana* solid phase microextraction results. Not only that, the essential oil of *L. pinceana* flowers might have special therapeutic qualities in view of the above active ingredients among the volatile compounds.

Benzenoids were the most abundant amongst floral scent compounds, which content reached at least 51%. The same scenario was noted in the floral essential oil of *Randia matudae* [17] compared to other species of different genera in the family Rubiaceae. In contrast, the quantity and amount of predominant compounds in the floral scent of *Posoqueria latifolia* [18], the leaf essential oil of *Rustia formosa* and the essential oil from aerial parts of *Anthospermum emirnense* and *A. perrieri* were sesquiterpenes [16,19]; the floral scent of *Cephalanthus occidentalis*, *Warszewiczia coccinea* and *Gardenia jasminoides* were monoterpenes [20,21]; the floral scent of *Coffea Arabica* were aliphatics [22]. It has been reported that the floral scent composition probably significantly varied amongst closely related species, and our results partly support this view [10].

### 2.2. Dynamic Changes of Scent Emission in Different Development Floral Stages

**Figure 1:**
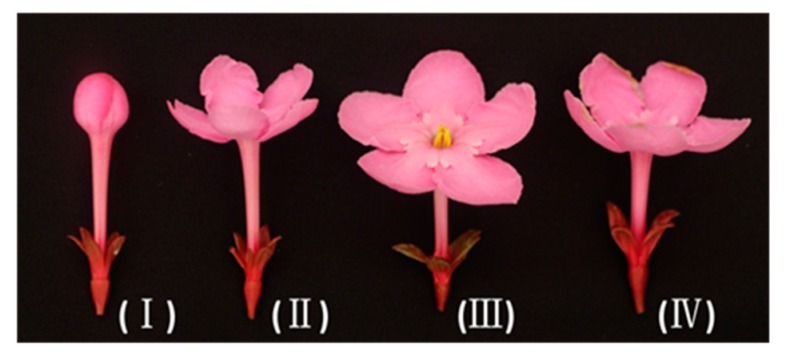
The morphological characteristics of *L. pinceana* flower in four different stages. (**I**) bud stage; (**II**) initial-flowering stage; (**III**) full-flowering stage; and (**IV**) end-flowering stage.

**Figure 2:**
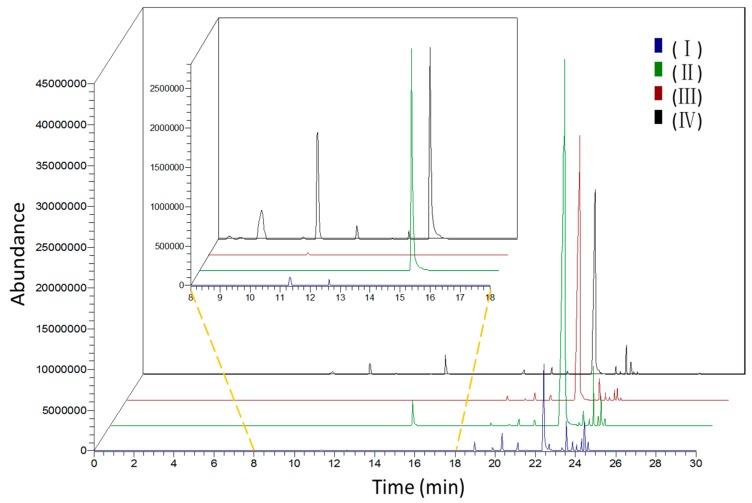
Total ionic chromatogram of volatile components emitted from flowers of *L. pinceana* in different stages. (**I**) bud stage; (**II**) initial-flowering stage; (**III**) full-flowering stage; and (**IV**) end-flowering stage.

*L. pinceana* flowers were selected on the basis of their botanical characteristics to evaluate the dynamic changes and diversity of floral volatiles according to different development stages: bud stage, initial-flowering stage, full-flowering stage, and end-flowering stage (Figure 1). Table 1 and Figure 2 show the distinct changes in scent composition and concentration across flowering stages. Scent components were drastically emitted at the initial-flowering stage, and then declined gradually at the full-flowering stage. The amount of VOCs at the bud stage and the end-flowering stage was obviously lower than that at the initial-flowering stage. The emission pattern of *L. pinceana* flowering stages was different from *Cananga odorata* [23], *Vanda* Mimi Palmer [24], and *Hosta* flowers [25] of which the fragrance ingredients were drastically emitted at the full-flowering stage and decreased greatly afterwards. These results showed that the emissions at different flower stages evidently differed. Investigation of the spatial and temporal patterns of gene expression has provided new information on the factors regulating the emission of plant volatile compounds [26].

As for the bud stage, 26 volatile compounds belonging to different chemical classes were identified: benzenoids (51.61%), sesquiterpenes (44.41%), aliphatics (2.46%), and monoterpenes (1.48%). The most abundant compound was paeonol, accounting for about 52% of the total GC peak area, followed by δ-cadinene (10.98%), cubebol (5.48%), isoledene (5.45%), and cyclosativene (4.96%). By contrast, relative content of (*E*)-β-ocimene (0.87%), (−)-β-cadinene (2.34%), cubebol (5.48%), and cubenol (1.02%) at the bud stage were higher than that at the other stages of flower development.

As for the initial-flowering stage, 26 volatile compounds belonging to different chemical classes were identified: benzenoids (85.88%), sesquiterpenes (13.37%), aliphatics (0.53%), and monoterpenes (0.15%). The most abundant compound was paeonol, accounting for about 83% of the total GC peak area, followed by (*E*,*E*)-α-farnesene (3.89%), methyl salicylate (2.85%), and δ-cadinene (2.61%). On the other hand, relative content of nonanoic acid, ethyl ester (0.01%) and methyl salicylate (2.85%) in the initial-flowering stage were significantly lower than that in the end-flowering stage, but was higher than that in the bud stage and the full-flowering stage. Relative content of hexyl caprylate was significantly lower than that in the other flower development stages.

As for the full-flowering stage, 32 volatile compounds belonging to different chemical classes were identified: benzenoids (80.05%), sesquiterpenes (16.79%), monoterpenes (1.63%), and aliphatics (1.46%). The most abundant compound was paeonol, accounting for about 80% of the total GC peak area, followed by γ-muurolene (5.86%), (*E*,*E*)-α-farnesene (4.90%), and cyclosativene (2.07%). In addition, relative content of β-ylangene (0.09%) and γ-muurolene (5.86%) in the full-flowering stage were higher than that in the other stages of flower development.

As for the end-flowering stage, 32 volatile compounds belonging to different chemical classes were identified: benzenoids (75.38%), sesquiterpenes (13.91%), monoterpenes (7.91%), and aliphatics (2.81%). The most abundant compound was paeonol, accounting for about 70% of the total GC peak area, followed by methyl salicylate (4.81%), (*E*,*E*)-α-farnesene (4.75%), and 3-carene (3.73%). By contrast, relative content of monoterpenes in the end-flowering stage were significantly higher than that in the other stages of flower development, including (1*S*)-2,6,6-trimethylbicyclo[3.1.1]hept-2-ene (0.45%), α-pinene (2.11%), 3-carene (3.73%), and α-santoline alcohol (0.32%). Relative content of nonanoic acid, ethyl ester (0.02%) and benzyl benzoate (0.78%) in the end-flowering stage were significantly higher than that in the other stages of flower development.

The Bray-Curtis similarity index is a statistic used for comparing the similarity of two samples [27]. The mean Bray-Curtis similarity index was 74.57% ± 11.53% (range: 61.81%~90.23%, *n* = 12, Table 2). The initial-flowering stage was more similar to the full-flowering stage (*BCS* = 90.23%) than to the end-flowering stage (*BCS* = 83.59%), and was largely dissimilar to the bud stage (*BCS* = 62.77%). Across all the flower-life stages, the bud stage was distinctly dissimilar to the full-flowering stage (*BCS* = 61.81%). Variations of the volatile compositions were apparently involved in the maturity stages of flower. The same phenomena are also observed in other plants, such as the flowers of *Ocimum citriodorum* [28], *Penstemon digitalis* [29], and *Cananga odorata* [23]. In this study, the highest diversity of floral volatiles was detected at the third and later periods of the flower development. Meanwhile, the richness of volatile compounds showed an unimodal pattern between the number of VOCs and times of flower development.

**Figure 3:**
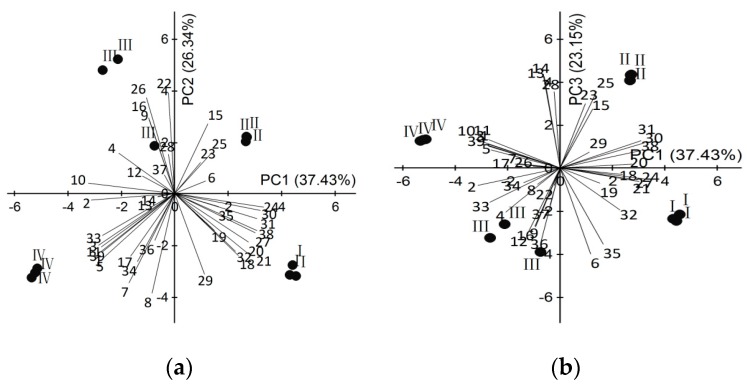
Principal component plot (PC1 *vs.* PC2 plots (**a**) and PC1 *vs.* PC3 plots (**b**)) for *L. pinceana* at different stages of growth, showing correlations with volatiles (numbers correspond to those in Table 1). (**I**) bud stage; (**II**) initial-flowering stage; (**III**) full-flowering stage; and (**IV**) end-flowering stage.

To identify which volatiles contributed the most to the differences among the four flower stages and to display the differences in a more visually appealing manner, the data on 39 volatile compounds identified in *L. pinceana* at a full life-flower scale were analyzed by using principal component analysis (PCA). The first three components of PCA explained 37.43%, 26.34%, and 23.15% of the variation, explaining ~87% of combined variance (Figure 3). Hereinto, volatiles that had high positive scores on PC 1 included (−)-β-cadinene, δ-cadinene, cadine-1,4-diene, cubenol, cubebol, isoledene, caryophyllene and α-cubebene, which were highly positively related to the bud stage and the initial-flowering stage. Volatiles with high positive scores on PC 2 comprised β-ylangene, γ-muurolene, unknown-1, unknown-2 and perilla alcohol, which were positively correlated with the full-flowering stage and initial-flowering stage. Volatiles with high positive scores on PC 3 included *cis*-verbenol, α-acorenol, (3*E*,5*Z*)-1,3,5-undecatriene and cedrol, which were negatively correlated with the full-flowering stage. The remaining 22 volatiles were composed of common components, megastigma-4,6(*E*),8(*E*)-triene, cyclosativene and 4-*epi*-cubebol. The principal component plots did not overlap amongst the four flower developmental stages indicated that the composition and its relative content of floral scent differed throughout the whole floral development, and the initial-flowering stage was recommended the best harvesting time when the high level of VOCs and essential oil are a concern.

## 3. Materials and Methods

### 3.1. Plant Materials

Fifteen fresh early flowering inflorescences from three *L. pinceana* plants (separation between plants more than 100 m, five inflorescences per plant) were collected from the Nujiang Lisu Autonomous Prefecture, Yunnan Province (25°58′N; 98°48′E) during their flourishing florescence on 10 November 2014, and inserted into deionized water before transported to the Research Institute of Resources Insects, Chinese Academy of Forestry (RIRICAF) in Kunming. Subsequently, the inflorescences were preserved at room temperature (25 ± 1 °C). The flowers were classified into four groups [23] according to their botanical characteristics (Figure 1): (I) bud stage: buds complete closed, two or three days before full bloom; (II) initial-flowering stage: semi-open petal, one day before full bloom; (III) full-flowering stage: completely open petals, observable pistils and stamens; and (IV) end-flowering stage: petals and calyxes withered, five or six day after full bloom.

### 3.2. Method

Volatile compounds of a complete flower were trapped immediately into a 20 mL capped solid-phase microextraction vial at 8:00–11:00 a.m. Three replicate experiments (five flowers from five inflorescences per replicate) were conducted using different plants randomly and the results were means of three tests (fifteen flowers).

SPME analysis was performed by 100 μm polydimethylsiloxane (PDMS) SPME fiber which was highly sensitive in the analysis of scent components equipped with a manual SPME holder. After the equilibration time, the fiber was exposed to the headspace of the capped vial to absorb volatile compounds for 40 min at room temperature (25 ± 1 °C). The fiber was conditioned in the GC injection port for 1 h at 250 °C before it was used for the first time. In addition, the empty capped vial was used as the blank control. A gas chromatograph-mass spectrometer (TRACE GC Ultra/ITQ900, Thermo Fisher Scientific, Waltham, MA, USA) coupled with a DB-5MS capillary column (5% diphenyl cross-linked 95% dimethylpolysiloxane, 30 m × 0.25 mm i.d., 0.25 μm film thickness. Agilent J & W Scientific, Folsom, CA, USA) were used for the GC-MS analysis. Follow the SPME analysis, the fiber with volatile compounds was exposed in the GC injector port for desorption at 260 °C for 1 min in the splitless mode. Helium was used as a carrier gas at a constant flow-rate of 1 mL/min. The oven temperature was programmed at 40 °C for 2 min, increasing 6 °C/min to 130 °C and then increasing 15 °C/min to 280 °C for 5 min. The split and splitless injection port were held at 260 °C and 200 °C in split mode at a split ratio of 1:10. The temperature of the transfer line and the ion source were 200 °C and 250 °C. The ionization potential of mass selective detector was 70 eV and the scan range was 50–650 amu.

### 3.3. Data Analysis

Identification of the volatile compounds was based on comparison of their mass spectra with NIST08 database through Software TF Xcalibur 2.1.0 (Thermo Fisher Scientific). Volatile compounds were identified on the basis of their linear retention index (*LRI*) and by comparing their mass spectra with a computerized MS-database using NIST 2008 library and published data (Pherobase, http://www.pherobase.com/; NIST, http://webbook.nist.gov/chemistry/; The LRI and Odour Database, http://www.odour.org.uk).

*LRI* were calculated by the use of a series of *n*-alkane standards (C6–C19) (Accu Standard, New Haven, CT, USA). It is defined as Equation (1):

(1) $$LRI=100\times n+\frac{100\times \left(t_{x}-t_{n}\right)}{t_{n+1}-t_{n}}$$

Peak areas were normalized as percentage and used to determine the relative amounts of the volatiles. The normalization method from the Equation (2):

(2) $$\text{Relative content}\;\left(\%\right)=\frac{\text{area under peak}}{\text{total peak area}}\times 100$$

The data were expressed as mean ± standard deviation (SD) of triplicate measurements. One-way analysis of variance (ANOVA) with Tukey’s test in SPSS software was used to assess differences in aroma compounds among four different stages of *L. pinceana* flower development. Volatile compounds identified at a full life-flower scale were analyzed by using principal component analysis (PCA) and Bray-Curtis similarity (BCS). PCA and BCS were carried out using PC-ORD for Windows (Version 5.0; MjM Software, Gleneden Beach, OR, USA).

## 4. Conclusions

The present investigation showed that 39 VOCs were identified in all flower-life stages of *L. pinceana*, amongst them, 26 at bud stage, 26 at initial-flowering stage, 32 at full-flowering stage, and 32 at end-flowering stage. Benzenoids were the most abundant amongst floral scent compounds. The amount of floral scent emission offered tendency of ascending first and descending in succession, reaching peak level at initial-flowering stage. The highest diversity of floral volatile was detected at the third and later periods of flower developmental stage. The odour evidently differed in composition and its relative content in the four flower developmental stages. The second flower developmental stage was recommended as the best harvesting time if a high level of VOCs and essential oil are of interest. *L. pinceana* could serve as beneficial houseplant in the future, besides as fragrant ornamental according to the VOCs emitted from the flowers. Furthermore, *L. pinceana* has a promising prospects for development as an essential oil source owing to the many medicinal properties of the main compounds of the floral scent.

## Figures and Tables

**Table 1:** Volatile compounds identified in four different stages of *L. pinceana* flower development using SPME-GC-MS. (**I**) bud stage; (**II**) initial-flowering stage; (**III**) full-flowering stage; and (**IV**) end-flowering stage.

Peak	RT	*LRI*	Compounds	CAS #	Relative Content(%) ± SD				
					I	II	III	IV
			**Monoterpenes**		**1.48 ± 0.69b**	**0.15 ± 0.02b**	**1.63 ± 0.83b**	**7.91 ± 1.15a**
1	8.37	904	(1*S*)-2,6,6-Trimethylbicyclo[3.1.1]hept-2-ene	7785-26-4	0b	0b	0b	0.45 ± 0.08a
2	8.75	918	Santolina triene	2153-66-4	0a	0a	0.32 ± 0.24a	0.34 ± 0.04a
3	9.46	943	α-Pinene	80-56-8	0b	0.05 ± 0.01b	0.26 ± 0.15b	2.11 ± 0.24a
4	10.84	994	Limonene	138-86-3	0a	0a	0.60 ± 0.43a	0.22 ± 0.03a
5	11.33	1012	3-Carene	13466-78-9	0.47 ± 0.48b	0.02 ± 0.00b	0.18 ± 0.12b	3.73 ± 0.61a
6	11.94	1034	(*Z*)-Verbenol	1845-30-3	0.05 ± 0.03a	0a	0.04 ± 0.02a	0a
7	12.37	1049	γ-Terpiene	99-85-4	0.03 ± 0.02a	0b	0b	0.03 ± 0.00a
8	12.64	1059	(*E*)-β-Ocimene	3779-61-1	0.87 ± 0.43a	0.02 ± 0.00b	0.09 ± 0.07b	0.61 ± 0.07ab
10	13.83	1102	α-Campholenal	91819-58-8	0b	0.02 ± 0.00b	0.07 ± 0.03a	0.08 ± 0.01a
11	14.36	1123	α-Santoline alcohol	90823-36-2	0.02 ± 0.01b	0.02 ± 0.00b	0.04 ± 0.03b	0.32 ± 0.06a
15	18.31	1295	Perilla alcohol	536-59-4	0.04 ± 0.02a	0.03 ± 0.00a	0.05 ± 0.02a	0.02 ± 0.00a
			**Aliphatics**		**2.46 ± 1.31a**	**0.53 ± 0.08a**	**1.46 ± 0.48a**	**2.81 ± 0.33a**
12	14.84	1142	(3 *E*,5Z)-1,3,5-Undecatriene	51447-08-6	0.04 ± 0.02a	0a	0.06 ± 0.04a	0.03 ± 0.00a
14	17.39	1251	Nonanoic acid,ethyl ester	123-29-5	0c	0.01 ± 0.00b	0c	0.02 ± 0.00a
17	18.98	1318	Megastigma-4,6(*E*),8(*E*)-triene	51468-86-1	2.13 ± 1.28a	0.48 ± 0.07a	1.18 ± 0.53a	2.49 ± 0.31a
33	25.40	1577	Hexyl caprylate	1117-55-1	0.08 ± 0.05ab	0b	0.08 ± 0.02ab	0.10 ± 0.01a
34	25.62	1592	Pentanoic acid, 2,2,4-trimethyl- 3-carboxyisopropyl, isobutyl ester	959016-51-4	0.22 ± 0.13a	0.04 ± 0.00a	0.14 ± 0.02a	0.17 ± 0.02a
			**Benzenoids**		**51.61 ± 22.32a**	**85.88 ± 2.45a**	**80.05 ± 6.24a**	**75.38 ± 3.47a**
13	15.08	1151	Methyl salicylate	119-36-8	0c	2.85 ± 0.45b	0c	4.81 ± 0.46a
23	22.43	1433	Paeonol	552-41-0	51.58 ± 22.36a	83.03 ± 2.91a	79.72 ± 6.14a	69.75 ± 4.00a
37	26.18	1639	Phenol, 2,6-bis(1,1-dimethylethyl)-4-(1-methylpropyl)	14035-34-8	0a	0a	0.30 ± 0.36a	0.04 ± 0.00a
39	27.75	1791	Benzyl benzoate	120-51-4	0.03 ± 0.04b	0b	0.03 ± 0.01b	0.78 ± 0.07a
			**Sesquiterpenes**		**44.41 ± 21.11a**	**13.37 ± 2.34a**	**16.79 ± 5.16a**	**13.91 ± 1.99a**
18	19.87	1346	α-Cubebene	17699-14-8	2.21 ± 1.42a	0.32 ± 0.06a	0.28 ± 0.26a	0.52 ± 0.07a
19	20.35	1362	Cyclosativene	22469-52-9	4.96 ± 2.66a	1.04 ± 0.15a	2.07 ± 1.17a	1.87 ± 0.16a
20	21.12	1386	Isoledene	95910-36-4	5.45 ± 3.16a	0.96 ± 0.20a	0.57 ± 0.73a	1.06 ± 0.12a
21	21.53	1399	Caryophyllene	87-44-5	1.34 ± 0.76a	0.17 ± 0.03a	0.15 ± 0.08a	0.23 ± 0.01a
22	21.89	1413	β-Ylangene	20479-06-5	0b	0.02 ± 0.00b	0.09 ± 0.03a	0b
24	23.37	1467	(-)-β-Cadinene	523-47-7	2.34 ± 1.35a	0.38 ± 0.06ab	0.58 ± 0.20ab	0.12 ± 0.01b
26	23.59	1476	γ-Muurolene	30021-74-0	0b	1.35 ± 0.22b	5.86 ± 1.95a	1.46 ± 0.14b
27	23.85	1485	Cubebol	23445-02-5	5.48 ± 3.14a	0.77 ± 0.11a	0.56 ± 0.63a	0.46 ± 0.08a
28	24.06	1493	(*E,E*)-α-Farnesene	502-61-4	2.46 ± 1.47a	3.89 ± 1.10a	4.90 ± 4.38a	4.75 ± 0.98a
29	24.30	1503	4- *epi*-Cubebol	23445-02-5	4.76 ± 2.65a	0.91 ± 0.15a	0.73 ± 0.82a	2.29 ± 0.30a
30	24.45	1513	δ-Cadinene	483-76-1	10.98 ± 6.25a	2.61 ± 0.10ab	0.64 ± 0.75b	0.60 ± 0.08b
31	24.62	1525	Cadine-1,4-diene	16728-99-7	3.19 ± 1.84a	0.77 ± 0.11a	0.25 ± 0.31a	0.42 ± 0.03a
35	25.69	1597	Cedrol	77-53-2	0.11 ± 0.06a	0a	0.05 ± 0.03a	0a
36	25.81	1606	α-Acorenol	28296-85-7	0.12 ± 0.07a	0a	0.07 ± 0.03a	0.05 ± 0.01a
38	26.33	1652	Cubenol	21284-22-0	1.02 ± 0.58a	0.18 ± 0.04ab	0b	0.07 ± 0.01b
			**Unknowns**		**0.03 ± 0.02ab**	**0.07 ± 0.01a**	**0.08 ± 0.03a**	**0b**
9	12.95	1070	Unknown-1	-	0b	0b	0.02 ± 0.01a	0b
16	18.60	1306	Unknown-2	-	0b	0b	0.05 ± 0.02a	0b
25	23.44	1470	Unknown-3	-	0b	0.07 ± 0.01a	0b	0b
32	25.27	1568	Unknown-4	-	0.03 ± 0.02a	0b	0b	0b

Values, expressed as mean ± SD of triplicate measurements, with different letters (a–c) in the same raw were significantly different according to Tukey’s test (*p* < 0.05). RT: retention time; *LRI*: linear retention index; CAS #: Chemical Abstracts Service Registry Number.

**Table 2:** The Bray-Curtis similarity index (%) among different stages of *L. pinceana* flower development. (**I**) bud stage; (**II**) initial-flowering stage; (**III**) full-flowering stage; and (**IV**) end-flowering stage.

	I	II	III	IV
**I**	100			
**II**	62.77	100		
**III**	61.81	90.23	100	
**IV**	65.32	83.59	83.71	100
